# Prevalence of Hepatitis C among Egyptian Children with Sickle Cell Disease and the Role of IL28b Gene Polymorphisms in Spontaneous Viral Clearance

**DOI:** 10.4084/MJHID.2016.007

**Published:** 2016-01-01

**Authors:** Somaia Mohammed Mousa, Mona Kamal El-Ghamrawy, Heba Gouda, Mervat Khorshied, Dina Abd El-Salam Ahmed, Hala Shiba

**Affiliations:** 1 Clinical Pathology Department, Kasr Al-Ainy School of Medicine, Cairo University, Cairo, Egypt.; 2 Pediatrics Department, Kasr Al-Ainy School of Medicine, Cairo University, Cairo, Egypt.

## Abstract

**Background and Objectives:**

Hepatitis C virus (HCV) is a major health problem in Egypt with its prevalence estimated to be 14.7% among the general population in 2008. Patients receiving frequent blood transfusions like those with sickle cell disease (SCD) are more exposed to the risk of acquiring HCV. IL28B gene polymorphisms have been associated with spontaneous HCV clearance. This study aims to determine the prevalence of HCV infection among children with SCD and to investigate the relation between IL28B gene polymorphisms and spontaneous HCV clearance.

**Methods:**

Seventy SCD patients were screened for HCV antibody. HCV-positive patients were tested for the level of HCV RNA using quantitative real-time PCR. IL28B polymorphisms (rs 12979860 SNP and rs 12980275 SNP) were detected using TaqMan QRT-PCR and sequence-specific primers PCR respectively.

**Results:**

Sixteen patients (23%) were HCV antibody positive, 9 of them (56.3%) had undetectable HCV RNA in serum, and 7 (43.7%) had persistent viremia. Genotypes CC/CT/TT of rs12979860 were found in 30 (42.9%), 29 (41.4%) and 11 (15.7%) patients and rs12980275 AA/AG/GG were found in 8 (11.4%), 59 (84.3%) and 3 (4.3%) patients. There was no significant difference in the frequency of IL28B (rs 12979860 and rs12980275) genotypes among HCV patients who cleared the virus and those with persistent viremia (p=0.308 and 0.724 respectively).

**Conclusion:**

Egyptian SCD patients have a high prevalence of HCV. Multi-transfused patients still exposed to the risk of transmission of HCV. IL28B gene polymorphismsare not associated with spontaneous clearance of HCV in this cohort of Egyptian children with SCD.

## Introduction

Egypt has the highest hepatitis C virus (HCV) prevalence worldwide.^1^ The prevalence of HCV in Egypt is found to be 14.7% among the general population in the year 2008.^2^ HCV prevalence is even higher among hospitalized patients and special clinical populations who have an increased risk of exposure to HCV like multi-transfused patients, thalassemic patients, and patients on hemodialysis.^3^ The prevalence of HCV among Brazilian sickle cell disease (SCD) patients was found to be 14%.^4^ Again, a study from the USA found that 22% of SCD patients were HCV-antibody positive. HCV positivity was most prevalent (58%) among patients whom blood were drawn before 1992 when testing of all blood donors in the USA became mandatory.^5^ To date, the prevalence of HCV among SCD patients in Egypt is not known.

During the natural course of HCV, about 15% of patients show spontaneous viral clearance without treatment.^6^ HCV spontaneous clearance was defined as the lack of HCV-RNA detection in the serum of the patient in the presence of a positive antibody response and absence of antiviral therapy.^7^ Factors affecting viral clearance include age, gender, race, level of viremia, alcohol intake, and HCV genotype. Genetic studies showed that single nucleotide polymorphisms (SNPs) in the IL28B gene, which encodes interferon (IFN)-λ-3 are associated with spontaneous HCV clearance.^8^ Among the most significant SNPs were rs12979860 and rs12980275.^9^–^11^ The aim of this study is to determine the prevalence of HCV infection and IL28B gene polymorphisms among Egyptian children with SCD and to explore the possible relation between IL28B SNPs (rs12979860 and rs12980275) and spontaneous viral clearance.

## Patients and Methods

### Patients

This cross-sectional study included 70 Egyptian children with SCD. The mean age of the patients was 10.2±4.5 years. They were 37 (52.8%) females and 33 (47.2%) males. Patients were consecutively invited to participate in the study during their regular follow-up visits at Pediatric Hematology Clinic, New Children Hospital, Cairo University. The study was approved by the Ethical Committee of Kasr Al-Ainy School of Medicine, Cairo University and patients were recruited after informed consents were freely obtained from their guardians.

In our resource-limited setting, SCD patients are not routinely or regularly screened for HCV. Therefore, during recruitment of the patients, their HCV status was not known, and none of the patients received antiviral treatment. Patients’ records were reviewed for the frequency of blood transfusion per year in the 12 months preceding the enrollment. Laboratory testing included complete blood count (CBC), reticulocyte count, Aspartate aminotransferase (AST) and Alanine aminotransferase (ALT) levels. Table 1 shows clinical and laboratory data of the patients. Patients were screened for HCV antibodies, and then quantification of HCV RNA in serum for patients who were HCV antibody positive was done. To estimate the frequencies of the IL28B genotypes in Egyptians, the SNPs rs12979860 C/T SNP and rs12980275 A/G SNP were genotyped in the whole group of SCD patients.

### Methods

Serum samples were tested for HCV antibody using a third generation enzyme immunoassay (EIA) (Ortho HCV 3.0 ELISA test system, Ortho Clinical Diagnostics Inc., Raritan, NJ, USA).

HCV RNA was quantified using a commercial real-time RT-PCR assay (RealTime™ HCV, Abbott Molecular Inc., Des Plaines, IL, USA) as specified by the manufacturer. The detection limit was 12 IU/mL.

For detection of IL28B polymorphisms, DNA was extracted using AxyPrep Blood Genomic DNA Miniprep Kit (Axygen Biosciences, USA). IL28B polymorphisms rs 12979860 C/T genotyping was performed using the ABI TaqMan allelic discrimination kit (Applied Biosystems, Foster City, California, USA) as described before.^12^

The sequence of the used primers were: Forward 5′-TGCCTGTCGTGTACTGAACCA-3′ and Reverse 5′-GAGCGCGGAGTGCAATTC-3′.

The sequences of the Taqman probes were: Probe for the C allele TGGTTCGCGCCTTC (VIC ™-labeled) and Probe for the T allele CTGGTTCACGCCTTC (FAM ™-labeled).

The PCR reaction was carried out in a total volume of 25μL. The following amplification protocol was used: pre-incubation at 50°C for 2 minutes and then 95°C for 10 minutes, followed by 40 cycles of denaturation at 95°C for 15 seconds, and annealing/extension at 60°C for 1 minute.

The sequence specific primer -PCR was applied for genotyping IL28B SNP rs12980275A/G polymorphism as described previously.^13^ A common reverse primer and two sequence-specific forward primers were used.

Gen (antisense) 5′-ATGATCATAGCTCATTGCAGC-3′ A allele-specific (sense) 5′-AGAAGTCAAATTCCTAGAAAC A-3′ G allele-specific (sense) 5′-AGAAGTCAAATTCCTAGAAAC G-3′. Cycling condition was denaturation for 1 minute at 95°C, followed by 46 cycles of amplification; denaturation for15 seconds at 95°C, annealing for 30 seconds at 50°C and extension for 30 seconds at 72°C. In the last cycle, the extension was prolonged to 7 minutes at 72°C. An amplification product of 393 bp was detected.

### Statistical Methods

Data were analyzed using Statistical Package for Social Sciences (SPSS) version 21. Numerical data were expressed as mean ±standard deviation (SD) and compared by student’s t-test. Qualitative data were expressed as frequency and percentage and compared by Chi-square test or Fisher’s exact test as appropriate. Odds ratio (OR) with its 95% confidence interval (CI) were used for risk estimation. All p-values are two-sided. P-values < 0.05 were considered significant.

## Results

Screening of SCD patients for HCV antibody revealed that 16 (23%) patients were positive for HCV antibodies. HCV antibody-positive patients had significantly higher AST and ALT levels (p= 0.002 and <0.001, respectively) compared to HCV antibody-negative patients.

HCV antibody-positive patients had a history of significantly more frequent blood transfusion compared to HCV antibody-negative patients (p= 0.003). Patients who were receiving a frequent blood transfusion (≥4times/year) had about 14-fold increased risk to be infected with HCV (Table 2).

Among HCV antibody-positive patients (n=16), 9 patients (56.3%) had undetectable HCV RNA in serum (spontaneously cleared) and 7 patients (43.7%) were non-cleared. HCV non-cleared patients had significantly higher AST and ALT levels (p= 0.012 and 0.023, respectively) compared to patients with spontanous virus clearance.

Genotyping of SCD patients forIL28B rs 12979860 C/T polymorphism revealed that; the wild type (CC) was detected in 30 patients (42.9%), heterozygous genotype (CT) was detected in 29 patients (41.4%), and the homozygous genotype (TT) was detected in 11 patients(15.7%). The frequency of the wild allele (C) was 0.64, and the frequency of the mutant allele (T) was 0.36.

Genotyping of SCD patients for IL28B rs12980275 A/G polymorphism revealed that; the wild type (AA) was detected in 8 patients (11.4%), heterozygous genotype (AG) was detected in 59 patients (84.3%), and the homozygous genotype (GG) was detected in 3 patients (4.3%). The frequency of the wild allele (A) was 0.54, and the frequency of the mutant allele (G) was 0.46. To analyze the impact of polymorphisms of IL28B gene on HCV clearance, the prevalence of rs12979860 C/T and rs12980275 A/G polymorphisms was compared between patients spontaneously clearing and patients not clearing HCV (Table 3), and no statistically significant difference was found between the two groups as regard prevalence of polymorphisms.

## Discussion

The prevalence of HCV among SCD patients in Egypt is not known. The present study is the first to investigate HCV prevalence among Egyptian SCD patients that is found to be 23%. Previous studies in Egypt revealed that HCV prevalence is high among all special clinical population groups like hemodialysis patients (35%),^14^ hemophilic children (40%),15 multi-transfused thalassemic patients (40.5%),^16^ non-Hodgkin’s lymphoma patients (43%)^17^ and hospitalized patients referred for bone marrow examination (42%).^18^ Hospitalization, repeated blood transfusions, invasive procedures, injections and shared dialysis machines are common risk factors for HCV infection among these group of patients. In this study, HCV antibody-positive patients received blood transfusion more frequently than HCV antibody-negative patient. Although the incidence of transfusion-acquired infections has significantly decreased in recent years because of more effective donor screening methods, the risk is still present, especially in multi-transfused patients.^19^ At Cairo University blood bank, donated blood is routinely screened for HCV using a third generation EIA. The more sensitive molecular methods utilizing nucleic acid amplification technology are not used due to limited resources. Previous studies have shown that the number of transfusions was directly correlated with the HCV antibody positivity. Patients who received 10 or more blood units had significantly higher incidence of anti-HCV markers than those receiving less than ten units of blood products.^5^ In the current study, patients who were receiving a frequent blood transfusion (≥4 times/year) had about 14-fold increased risk to be infected with HCV.

Hepatitis C leads to persistent infection in a high proportion of infected individuals, and can progress to chronic liver disease, cirrhosis, and hepatocellular carcinoma. HCV spontaneous clearance was defined as the lack of HCV-RNA detection in the serum of the patient in the presence of a positive antibody response and absence of antiviral therapy.^7^ In the current study, 56.3% of HCV positive patients spontaneously cleared the virus. During the natural course of HCV, about 15% of patients showed spontaneous viral clearance without treatment.^6^ As observed in the current study, higher rates of spontaneous resolution have been found in children. In a prospective study including 67 patients with chronic HCV, infection due to blood transfusion at a mean age of 2.8 years, the infection resolved in 30 patients (45%) after a mean follow-up of 20 years; of the remaining 37 patients, only 1 had abnormal liver enzymes, and only 3 showed signs of histologic damage.^20^ Factors responsible for age-related differences in the clinical course of chronic HCV infection may include, structural and/or immunologic differences between children and adults including less availability of antioxidizing agents and fat infiltration of adult liver.^21^

Ethnic differences in the frequency of virus clearance suggest that host genetic variation may have an impact on HCV clearance.^22^ Genetic studies showed that genetic variation in the IL28B gene, which encodes IFN-λ-3 is associated with spontaneous HCV clearance.^8^ Other studies have reported associations of SNPs in IL28B gene with response to antiviral therapy. Among the most significant SNPs were rs12979860 and rs12980275.^9^–^11^ In the current study, the allele frequency at the rs12979860 SNP was 64% for the wild allele C and 36% for the mutant allele T. The frequency of the wild allele in this study is similar to that detected in a previous Egyptian study (67%),^23^ in another North African (Moroccan) population (68%)^12^ and in some European populations (60–70%),^8^ but higher than that found in southern African populations (23–40%) and less than in Asian population (75–98%).^8^ The allele frequency at the rs12980275 SNP was 54% for the wild allele A and 46% for the mutant allele G. This wild allele frequency is similar to that among Caucasian population (52%)^13^ but less than that detected in previous studies including Saudi population (62%)^24^ and European population (62.6%).^25^

Our results show that IL28B gene polymorphisms are not associated with spontaneous resolution of HCV in this group of children with SCD. However, the generalization of these preliminary observations is limited by the small sample size. The association between SNP (rs12979860) of the IL28Bgene and the outcome of HCV infection was first described in 2009. Investigators found that the CC genotype of the rs12979860 SNP was associated with an improved response to treatment of adult patients with HCV independent of their ethnicities.^9^ Since that, multiple studies have confirmed and reinforced the association between the C allele of the rs12979860 SNP and both spontaneous and treatment-induced clearance of HCV in adults.^26^

Fewer data are available regarding the effect of rs12980275 SNP of IL28B gene on HCV outcome. A genome-wide association study in 2009 found that the A allele of the rs12980275 SNP was associated with good response to treatment in adult Japanese patients with HCV.^11^ A subsequent study confirmed the association between rs12980275 SNP and higher response to treatment in adults with HCV.^24^

In children, data about the relation between polymorphisms in IL28B gene (rs12979860 and rs12980275) and spontaneous clearance of HCV is limited. In contrast to our results and in agreement with that already demonstrated in adults, two studies published in 2011 demonstrated for the first time that the C allele of rs12979860 SNP of the IL28B is associated with spontaneous clearance of HCV in children.^27^,^28^ These preliminary results have been confirmed in a subsequent study including a larger cohort of Italian children with HCV.^29^

To our knowledge, no previous study addressed the relation between rs12980275 SNP of IL28B gene and spontaneous resolution of HCV in children.

The mechanism behind the association of genetic variations in the IL28Bgene and spontaneous clearance of HCV may be related to the host innate immune response. IL28B encodes IFN-λ-3, which is involved in viral control, including HCV.^30^ Both IFN-α and IFN-λ-3 bind to cell-surface receptors leading to induction of interferon stimulating genes, a mechanism by which IFNs suppress viral infections.^30^–^32^

## Conclusions

the present study revealed that the prevalence of HCV infection among Egyptian patients with SCD is considerably high. Despite more-effective donor screening methods, blood transfusion still carries a risk of transmission of HCV, especially in multi-transfused patients. Efforts should be made to implement a more sensitive molecular method for screening of blood products for HCV. In the current study, more than half of HCV positive patients show spontaneous viral clearance. Polymorphisms in the IL28B may not be associated with spontaneous clearance of HCV infection among this group of children with SCD. This study is limited by the small number of HCV positive patients. Before generalization of our results, larger studies are required to assess better the impact of genetic variation in IL28B gene on HCV outcome in children.

## Figures and Tables

**Table 1:** Clinical and laboratory data of sickle cell disease patients (n=70).

Variable	No (%) or mean±SD

Blood transfusion frequency:	
Frequent (≥4 times/year)	43(61.4)
Infrequent (1 – 3 times/year)	6(8.6)
Once/life	5(7.1)
Never	16(22.9)

Hemoglobin (g/dL)	8.0±1.2

White blood cells (×10^3^/μL)	9.0±2.8

Absolute neutrophil count	4493±1789

Platelets (×10^3^/μL)	354±156

Alanine aminotransferase (IU/L)	29.3± 53.2

Aspartate aminotransferase (IU/L)	44.6±47.6

**Table 2:** Comparison between HCV antibody-positive and HCV antibody-negative patients as regard frequency of blood transfusion, values are expressed as n (%).

Criteria	HCV-negative n=54	HCV-positive n=16	P value	Odds ratio (95% CI)
Frequent blood transfusion	28(52)	15(94)	0.003	13.9 (1.7–113)
Infrequent blood transfusion/Once/Never	26(48)	1(6)		

**Table 3:** Comparison between HCV cleared and HCV non-cleared patients as regard prevalence of IL28 B rs12979860 C/T and rs12980275 A/G polymorphisms.

Genotype	HCV cleared n= 9	HCV non-cleared n=7	P value

IL28B rs12979860			
Wild (C/C)	4	1	0.308
Mutant (C/T, T/T)	5	6	

IL28B rs12980275			
Wild (A/A)	0	0	0.724
Mutant (A/G, G/G)	9	7	
